# A Novel Approach to Botulinum Toxin Injection for Neurogenic Detrusor Overactivity in Children

**DOI:** 10.7759/cureus.87034

**Published:** 2025-06-30

**Authors:** Antonio Marte

**Affiliations:** 1 Department of Woman, Child and General and Specialized Surgery, University of Campania, Naples, ITA

**Keywords:** children, neurogenic bladder, neurogenic detrusor overactivity, onabotulinum a, overactive bladder

## Abstract

Botulinum toxin (BoNT) injections into the bladder wall have become a well-established treatment for various bladder disorders, particularly neurogenic detrusor overactivity (NDO) and overactive bladder (OB) in both adults and children. However, challenges persist with injection techniques, as up to 40% of BoNT can inadvertently be injected into the extravesical space, leading to reduced treatment efficacy. There are two primary methods for administering BoNT: submucosal and intravesical injections, each employing distinct techniques to ensure precise drug delivery. A novel technique referred to as mucosal lifting was evaluated in 20 young patients with NDO. This method is a modification of the traditional submucosal injection technique, where the bladder mucosa is lifted to create a suitable space for injection. Results demonstrated a significant increase in dryness time between clean intermittent catheterizations, with an average improvement from 8.9 months to 11.8 months, indicating enhanced efficacy. Despite these encouraging findings, the small sample size limits the generalizability of the study's conclusions.

## Introduction

Botulinum toxin A (BoNT-A) has emerged over the past two decades as a potent therapeutic agent for various lower urinary tract dysfunctions, particularly in patients suffering from neurogenic detrusor overactivity (NDO) and overactive bladder (OAB). These conditions, often secondary to spinal dysraphism or other neurologic impairments, are characterized by involuntary detrusor contractions during the bladder filling phase, leading to urinary incontinence, elevated bladder pressures, and potential upper urinary tract damage. Transurethral injections of botulinum toxin into the bladder wall have become an integral part of the therapeutic arsenal for various bladder disorders, notably neurogenic detrusor overactivity and overactive bladder in both adults and children.

According to some authors, the current injection technique allows for an average of about 18% to as much as 40% of botulinum toxin to be inadvertently injected into the extravesical space, leading to reduced efficacy or lack of response [1]. Additionally, a shallow needle insertion may cause partial reflux of the toxin into the bladder cavity. Samal et al. evaluated intraluminal loss of botulinum toxin by adding methylene blue to reconstitute the toxin. They demonstrated minimal loss of toxin in the irrigation fluid [2]. This issue is often underestimated, yet it can significantly impact both the duration and effectiveness of the botulinum injection. Two main strategies for BoNT-A delivery into the bladder wall have been explored: intradetrusor and suburothelial injections. While both approaches show similar clinical effectiveness, the ideal injection technique should ensure maximal drug retention within the target tissue, avoid mucosal reflux, and account for the anatomic variability of the pediatric bladder. Most authors agree that the therapeutic effects and duration of intradetrusor versus suburothelial injections of onabotulinumtoxinA in patients with NDO are comparable [3]. Thus, the primary challenge lies in optimizing treatment effectiveness while minimizing the incidence of ineffective injections.

Moreover, regardless of whether submucosal or intravesical injection is chosen, patients with neurogenic bladder dysfunction or overactive bladder typically exhibit irregular bladder wall, characterized by trabeculations, wall thickening, and decreased compliance, compared to healthy individuals, which complicates the selection of the most suitable injection technique [4,5]. In 2009, Mehnert et al. were the first to demonstrate that injections can occasionally penetrate the extravesical space, potentially diminishing treatment efficacy [6]. Additionally, there is a risk that the botulinum toxin may disperse within the bladder, further compromising its therapeutic effect [3]. Given these considerations, novel refinements in injection methodology are necessary to enhance therapeutic outcomes. One such innovation is the “mucosal lifting” technique, a modified submucosal approach designed to optimize toxin placement by creating a visible and controlled mucosal bleb prior to injection. This technique may reduce drug loss, improve injection precision, and ultimately extend the duration of clinical benefit. In this study, we evaluated the feasibility and effectiveness of this approach in a pediatric cohort with neurogenic detrusor overactivity.

## Technical report

Since January 2022, we have implemented a modified approach to submucosal injection known as the mucosal lift technique. This case series comprises 20 patients aged seven to 17 years (mean age: 14) diagnosed with NDO. These patients had previously undergone one or more treatments using the conventional submucosal method developed by our group, which utilized a flexible Cook’s needle [7]. The duration of efficacy for these injections ranged from seven to 11 months, with a mean duration of 8.9 months.

This study was conducted in accordance with the Helsinki Declaration. All patients received an extratrigonal multipoint injection of 200 IU of onabotulinumtoxinA, diluted in saline to deliver 1 ml containing 10 IU of the toxin, in compliance with FDA pediatric guidelines [8]. In this novel technique, we employed a 25G metal needle, typically used for the endoscopic treatment of vesicoureteral reflux (VUR), rather than a flexible needle. The needle was inserted 4 to 5 mm into the bladder wall during moderate bladder filling. We then lifted both the needle and the cystoscope 2 to 3 mm, elevating the mucosa and creating a visible colliculus. This maneuver facilitated easy injection and ensured proper dispersion of the toxin into the submucosal space. If the needle was inserted too deeply and mucosal elevation was not achieved, the puncture was corrected by withdrawing the needle and repeating the process to ensure optimal administration. To facilitate the injection at the bladder dome, patients were positioned in a slight Trendelenburg posture. A catheter was placed for four to five hours post-procedure, with all patients discharged on the same day. No significant complications were reported, aside from occasional hematuria, and no notable side effects were observed. Given their prior uneventful treatments, immediate tolerability was reliably assessed. Moreover, patients were contacted within seven to 10 days post-procedure, and no adverse events were reported. The mean dry time between clean intermittent catheterizations (CIC) was compared before and after the introduction of the mucosal lift technique. Given the preliminary nature of this study and the limited sample size, we opted for a statistical analysis using the Student’s t-test, which revealed a significant increase in the mean dry interval, from an average of 8.9 months to 11.8 months (P < 0.0297) (Figure 2).

**Figure 1:**
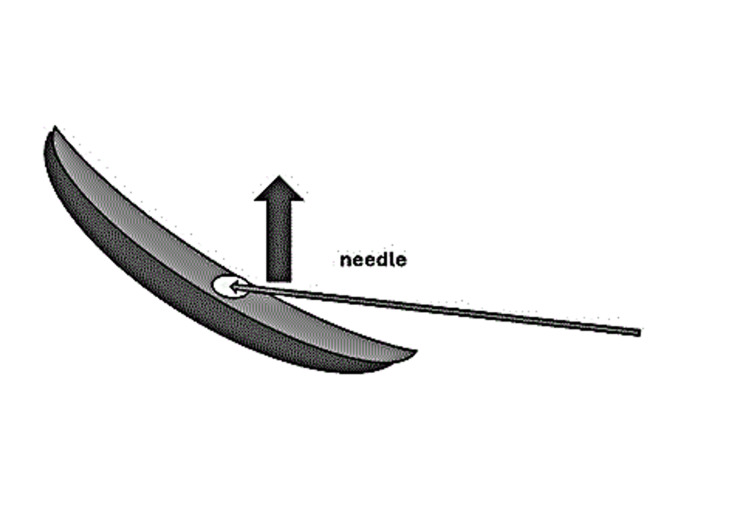
Diagrammatic representation of the injection technique The diagram illustrates the submucosal plane targeted by the needle, emphasizing the controlled lifting of the mucosa prior to injection. Original illustration created by the author.

**Figure 2:**
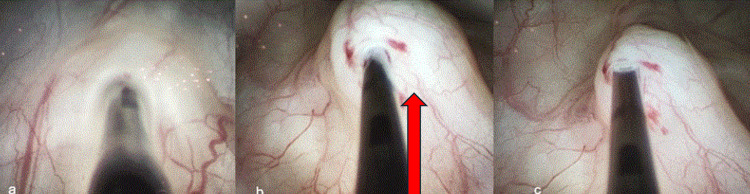
Bladder mucosal lifting technique used in botulinum toxin injection a) Insertion of the injection needle into the bladder mucosa using a cystoscopic approach. (b) Lifting of the mucosa to create a submucosal bleb (arrow), followed by precise injection of botulinum toxin. (c) Withdrawal of the needle after confirmed delivery of the toxin within the submucosal plane. All images are original and created by the author.

## Discussion

The success of botulinum toxin (BoNT) therapy in treating neurogenic detrusor hyperactivity (NDO) strongly depends on the precision of the injection technique. As evidenced by both clinical experience and existing literature, the variability of bladder wall thickness, particularly in pediatric patients, is a significant challenge. Bladder morphology can vary not only between individuals but also within the same patient over time due to factors such as bladder compliance, capacity, and the presence of detrusor fibrosis. Even when ultrasound monitoring is routinely used, bladder wall thickness may fluctuate, influencing needle depth and the accuracy of drug delivery. These anatomical differences necessitate an adaptable and patient-specific approach to BoNT injection. A standard technique may not adequately address these variations, especially in children whose bladders are smaller and more compliant. There remains a documented risk of either penetrating the full thickness of the bladder wall and injecting extravesically or, conversely, delivering the toxin too superficially into the intraluminal space. Both scenarios compromise the therapeutic effect of the treatment and likely contribute to the interpatient variability in clinical outcomes.

The importance of refining the technical aspects of BoNT injections was clearly emphasized by Karsenty et al., who conducted a comprehensive review of procedural elements, from the number and placement of injection sites to needle gauge and length [9]. Their recommendations aim to standardize the procedure, particularly in adults, to reduce technical failures and improve safety. However, it is worth noting that many of these technical details are based on data from adult studies and may not be directly applicable to pediatric populations. For example, a needle length of 4 mm with a stopper is suggested to avoid over-penetration, yet in a child’s bladder, even this depth may risk breaching the detrusor entirely if wall thickness is not carefully assessed intraoperatively. Our modified technique, the mucosal lift, was developed precisely to meet these needs. By elevating the bladder mucosa before toxin injection, we create a submucosal space that visually confirms adequate depth and helps to localize the agent within the bladder wall, minimizing both reflux and extravesical leakage. This technique showed promising results in our limited cohort, with a notable increase in the duration of dryness between catheterizations, suggesting improved efficacy.

Nonetheless, this study has clear limitations. The small number of patients reduces statistical power and prevents broad generalization of results. Moreover, the observed improvement over time could be influenced by other factors such as repeated BoNT injections or increasing operator experience. While a learning curve cannot be completely excluded, it is important to consider that all procedures in this series were performed by the same experienced surgeon. Furthermore, the literature suggests that the efficacy of repeated BoNT treatments tends to plateau rather than increase over time and often requires dose adjustment or site repositioning to maintain therapeutic benefit [10,11].

## Conclusions

Although our findings are based on a small cohort and clinical parameters, they suggest a promising trend toward improved therapeutic outcomes. Future developments in BoNT therapy will likely be influenced by two main factors: the availability of longer-acting formulations and further refinement of minimally invasive delivery methods.
